# Resection and postoperative radiation therapy for desmoid fibromatosis of the chest wall in a young woman

**DOI:** 10.1186/s40792-020-01006-5

**Published:** 2021-01-20

**Authors:** Daiki Noda, Miyuki Abe, Yohei Takumi, Kentaro Anami, Michiyo Miyawaki, Hideya Takeuchi, Atsushi Osoegawa, Kenji Sugio

**Affiliations:** Department of Thoracic and Breast Surgery, Faculty of Medicine, Oita University, 1-1 Idaigaoka, Hasama-machi, Yufu, Oita 879-5593 Japan

**Keywords:** Desmoid fibromatosis, Chest wall tumor, Chest wall resection, Adjuvant radiation therapy

## Abstract

**Background:**

Surgery is an effective treatment for desmoid fibromatosis, but it may be difficult, depending on the location or local spread of the tumor, and the decision to perform surgery must be made carefully. We herein report a case of desmoid fibromatosis of the chest wall in a young woman suspected of having invasion to the 1st, 2nd and 3rd ribs.

**Case presentation:**

A 35-year-old woman had been aware of dry cough and right chest pain, so she was referred to our hospital. Chest computed tomography showed a localized pleural tumor mainly at the first rib. Magnetic resonance imaging revealed a 75 × 65 × 27-mm tumor with a smooth surface, with partial contact from the first rib to third rib and partial extension to the 1st intercostal space. The tumor showed growth in the two months after the first visit, so resection was performed. The tumor was completely resected, and adjuvant radiation therapy (50 Gy) was performed for the small margin. The pathological diagnosis was desmoid fibromatosis. The postoperative course has been uneventful, without recurrence at 14 months after surgery.

**Conclusions:**

In chest wall tumors located ventral of the pulmonary apex, we suggest that a combination of the Grunenwald method and Masaoka anterior approach may be a useful option. In cases where margin is not enough, adjuvant radiation therapy should be considered.

## Background

Desmoid fibromatosis are myofibroblastic neoplasms of muscle aponeurosis origin that arise from fascial or deep musculoaponeurotic structures. Surgery is still an effective treatment for desmoid tumors but may be difficult, depending on the location or local spread of the tumor. Furthermore, desmoid fibromatosis is locally aggressive and associated with high recurrence rates after surgical resection, with some studies reporting recurrence rates of 20% to 70% [1]. In the past, surgery was actively performed, but recent guidelines state that it is necessary to carefully determine whether or not to perform surgery [2].

Chest wall resection including the 1st rib requires an ingenious approach due to the complex anatomical characteristics. Cosmetic management is also necessary for young women. We herein report a case of desmoid fibromatosis of the chest wall in a young woman suspected of invasion to the 1st, 2nd and 3rd ribs.

## Case presentation

**Fig. 1:**
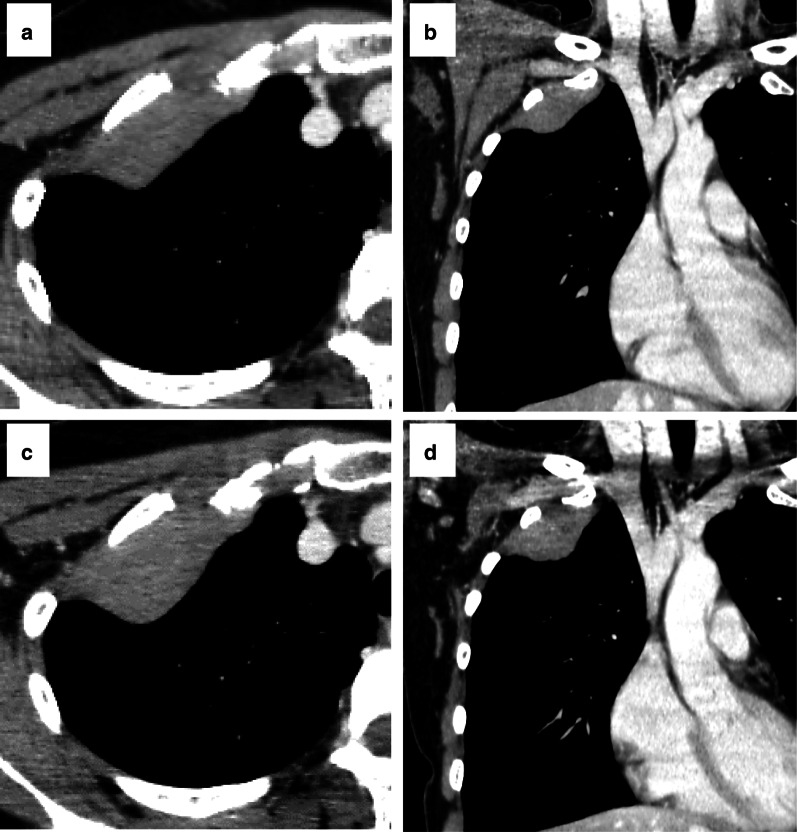
CT at the first visit. **a** Axial view. **b** Coronal view. There was a limbic smooth mass in extensive contact with the right chest wall. It had extended to the first and second intercostal area. No invasion to the subclavian vessels or brachial plexus was observed. CT two month after the first visit. **c** Axial view. **d** Coronal view. The tumor showed growth without invasion to surrounding tissues

A 35-year-old woman who had smoked 5 pack-years had been aware of dry cough and right chest pain for 2–3 years, and pain in the right upper limb had gradually appeared. Her laboratory data revealed anemia (Hb 10.0 g/dl). Chest X-ray demonstrated a mass in the right thorax. Chest computed tomography (CT) showed localized pleural tumor mainly at the first rib (Fig. 1a, b). Magnetic resonance imaging (MRI) revealed a 75 × 65 × 27-mm tumor with a smooth surface from the first rib to third rib and extending to the 1st intercostal space. Most of the mass was hyperintense on T2-weighted imaging and hypointense on T1- and diffusion-weighted imaging. On dynamic contrast-enhanced MRI, most of the mass was enhanced in the delayed phase. These findings were suggestive of solitary fibrous tumor, desmoid fibromatosis and neurogenic tumors originating from the intercostal nerves. CT showed growth in the two months after the first visit but no infiltration of the apex area or thoracic outlet (Fig. 1c, d). An invasive soft tissue tumor, such as solitary fibrous tumor or desmoid fibromatosis, was suspected, so resection of the tumor was planned.

**Fig. 2:**
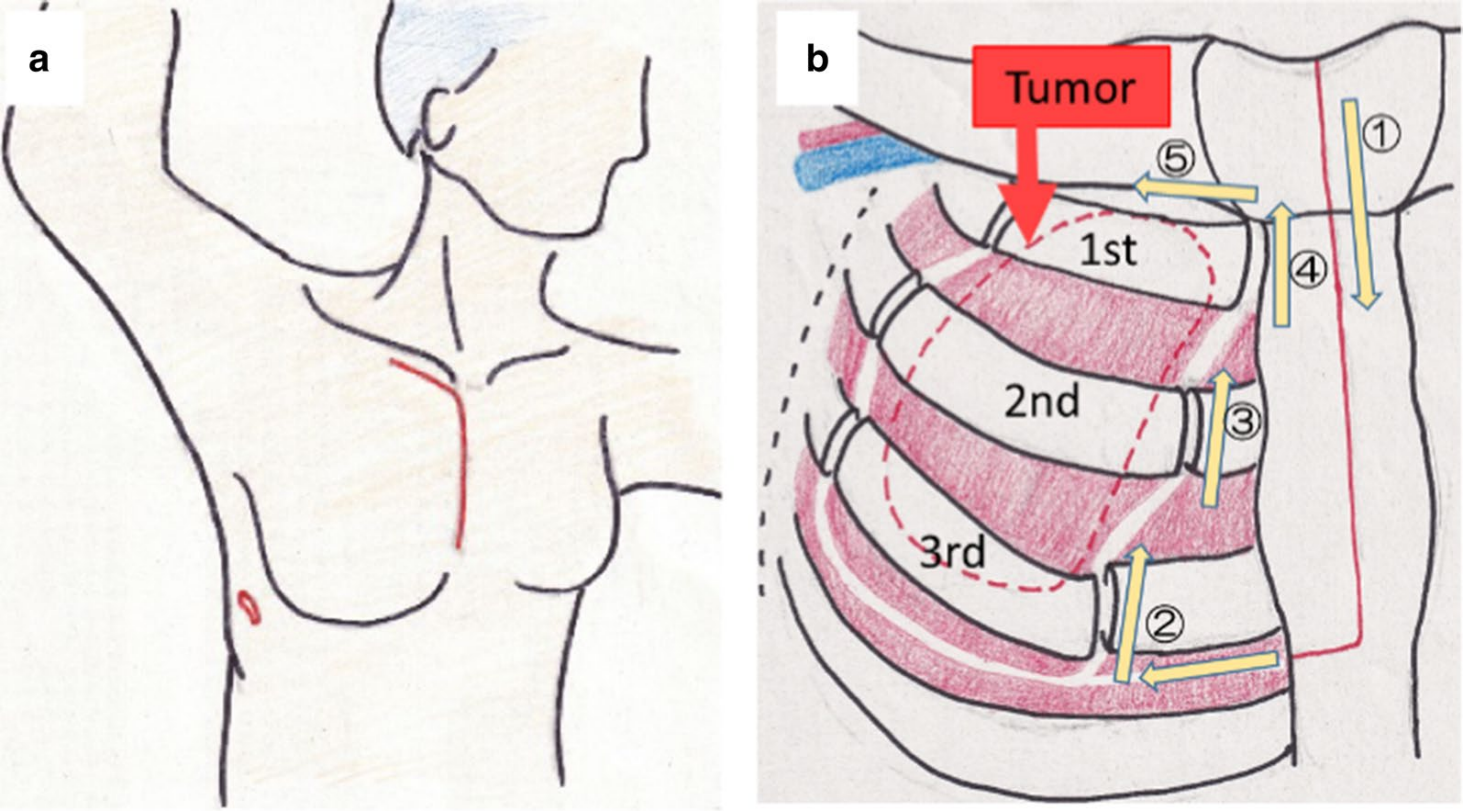
Schematic illustration of the position (**a**) and operative field (**b**). In the left hemi-lateral position, a incision was made below the right clavicle, and median sternotomy was made at the third intercostal position. Right hemisection of the sternum was performed, and the third intercostal space was opened

The patient underwent chest wall resection in the left hemi-lateral position. A thoracoscope was inserted into the right thoracic cavity, and the tumor was confirmed. A subclavian incision and median sternotomy to the third rib were made, and the third intercostal space was opened. The first rib was cut at the sternal attachment portion. The subclavian artery was confirmed, a sufficient distance from the tumor was identified. The major and minor pectoral muscles were released from the chest wall, and the three ribs (1st, 2nd, 3rd rib) were cut at the distal portion (Fig. 2). The tumor, including the three ribs, was then resected. The defective chest wall was covered with GORE-TEX® Dual Mesh 15 × 7 cm and sutured to the 4th rib and sternum. The operation time was 298 min, and the blood loss was 210 ml. The resected specimen was 17.5 × 7.5 cm. There was no flail chest after the surgery.

**Fig. 3:**
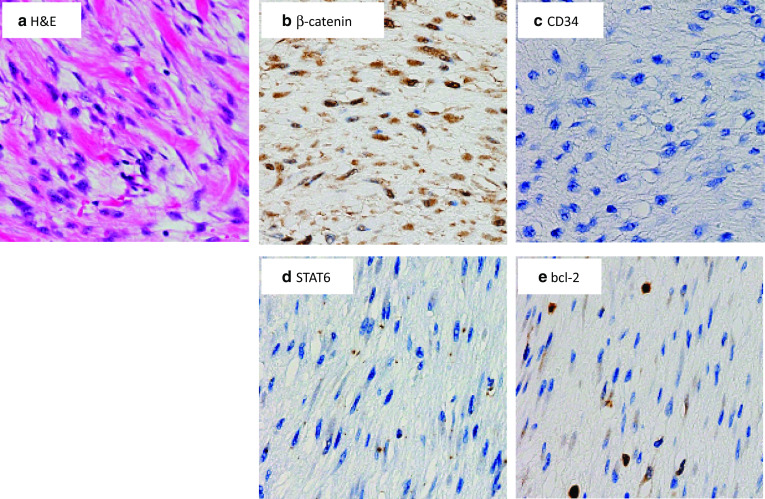
Pathological findings of the tumor. a. Spindle cells embedded in collagenous matrix were observed (**a** H&E). On immunohistochemistry, β-catenin (**b**) was positive. CD34 (**c**) and STAT6 (**d**) were negative, and bcl-2 (**e**) was slightly positive

Histopathologically, the tissue consisted of spindle cells embedded in a collagenous matrix (Fig. 3). Immunohistochemistry demonstrated negative staining for STAT6, CD34, and slightly positive staining for bcl-2. Vimentin and β-catenin were positive. These results suggested desmoid fibromatosis. The pathological margin was negative.

**Fig. 4:**
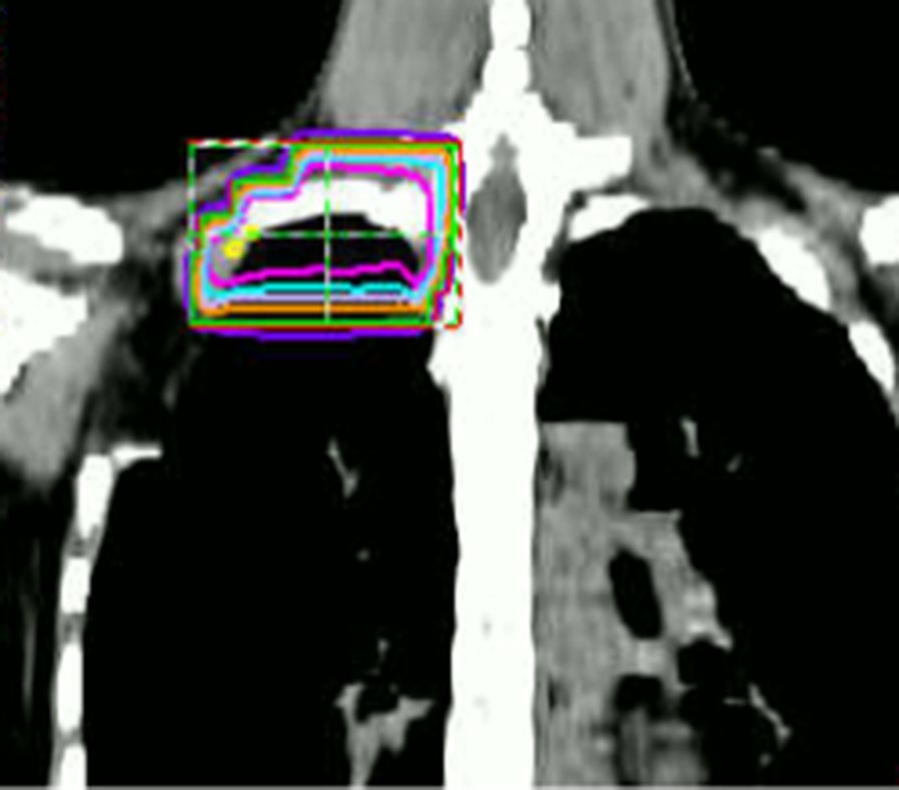
Radiation field: Adjuvant radiation therapy (50 Gy in 25 fractions) was performed postoperatively because there was a small margin on the tumor head side

The patient was discharged on the 16th day after surgery. The macroscopic surgical margin on the cranial side of the first rib was not considered sufficient, although the pathological margin was negative, Therefore, adjuvant radiation therapy (50 Gy) was performed (Fig. 4). The radiation field was selected carefully so as to minimize the dose to the lung and heart, and therefore radiation therapy was deemed to be performed safely. The postoperative course has been uneventful, without recurrence at 14 months after surgery.

## Discussion

In the WHO Classification of Tumors, desmoid tumors are defined as a myofibroblastic proliferative soft tissue tumors and classified as an intermediate malignancy. The incidence in the general population is two to four per million people per year. Desmoid tumors are more common in patients 15–60 years old and more frequent in women than in men. The origin is typically intra- or extra-abdominal, such as the abdominal wall, shoulder, breech, or limbs. Extra-abdominal desmoid tumors have been attributed to β-catenin gene mutations.

Surgery with extensive resection has been considered the major treatment, but the extremely high postoperative recurrence rate suggests the feasibility of nonsurgical treatments, as recommended by the European Organization for Research and Treatment of Cancer [3]. The Japanese Orthopaedic Association have proposed their own extra-abdominal desmoid type fibromatosis treatment guideline [2]. When tumor enlargement is observed or any symptoms occur, surgical resection is recommended in cases where the postoperative dysfunction is expected to be low. In the present case, during two months of follow-up, we noted a growing trend, and the possibility of malignancy could not be ruled out. When a tumor is located at the pulmonary apex and expected to invade the subclavian vessels and brachial plexus in the future, early resection is desirable.

Regarding the operative procedure in the present case, the modified Grunenwald method [4] and Trap-Door method [5] were performed, involving a subclavian incision and superior partial sternotomy to the third rib followed by opening the third intercostal space. This technique provides excellent exposure of tumors located in the anterior part of the lung apex. This allows for the safe exposure of the subclavian vessels and brachial plexus. In addition, a posterior field of view in combination with thoracoscopy can provide good exposure of the extent of the tumor.

Some studies have suggested that adjuvant radiation may be useful for managing desmoids. This approach is particularly effective in cases of microscopically positive resection margins [6, 7] or recurrent desmoid tumor [8]. Adjuvant radiation has shown an improvement in the recurrence-free survival in radiated microscopically margin-positive patients. In the present case, the tumor was located in a complex dissection of the chest wall, and there was a small margin between the subclavian vessels, brachial plexus, and sternocostal joint of the 1st rib. If recurrence were to occur, additional resection of the thoracic outlet area was expected to be difficult. Therefore, adjuvant radiation was performed. Surgery in a complex dissection is preferable to control the tumor while preserving the function through a combination of less burdensome resection and adjuvant radiation.

## Conclusion

In resection of chest wall tumors close to the pulmonary apex, due to complex anatomy, the approach requires an ingenuity. In such cases, a combination of the Grunenwald method and Masaoka anterior approach is recommended. When the margin is not enough, adjuvant radiation therapy should be considered.

## Data Availability

Data sharing is not applicable to this article as no datasets were generated or analyzed during the current study.

## Abbreviations

CT: Computed tomography
MRI: Magnetic resonance imaging
T2WI: T2-weighted imaging
T1WI: T1-weighted imaging
STAT6: Signal transducer and activator of transcription 6
CD34: Cluster of differentiation 34
Gy: Gray
WHO: World Health Organization
EORTC: European Organization for Research and Treatment of Cancer
